# Altered Causal Coupling Pathways within the Central-Autonomic-Network in Patients Suffering from Schizophrenia

**DOI:** 10.3390/e21080733

**Published:** 2019-07-26

**Authors:** Steffen Schulz, Jens Haueisen, Karl-Jürgen Bär, Andreas Voss

**Affiliations:** 1 Institute of Innovative Health Technologies, University of Applied Sciences, 07745 Jena, Germany; 2 Institute of Biomedical Engineering and Informatics, University of Technology, 98693 Ilmenau, Germany; 3 Department of Psychiatry and Psychotherapy, Pain and Autonomics-Integrative Research, University Hospital, 07745 Jena, Germany

**Keywords:** central-autonomic coupling, partial directed coherence, transfer entropy, schizophrenia, 87.18.-h, 87.85.-d, 87.85.Ng, 87.85.fp, 89.70.Cf

## Abstract

The multivariate analysis of coupling pathways within physiological (sub)systems focusing on identifying healthy and diseased conditions. In this study, we investigated a part of the central-autonomic-network (CAN) in 17 patients suffering from schizophrenia (SZO) compared to 17 age–gender matched healthy controls (CON) applying linear and nonlinear causal coupling approaches (normalized short time partial directed coherence, multivariate transfer entropy). Therefore, from all subjects continuous heart rate (successive beat-to-beat intervals, BBI), synchronized maximum successive systolic blood pressure amplitudes (SYS), synchronized calibrated respiratory inductive plethysmography signal (respiratory frequency, RESP), and the power P_EEG_ of frontal EEG activity were investigated for 15 min under resting conditions. The CAN revealed a bidirectional coupling structure, with central driving towards blood pressure (SYS), and respiratory driving towards P_EEG_. The central-cardiac, central-vascular, and central-respiratory couplings are more dominated by linear regulatory mechanisms than nonlinear ones. The CAN showed significantly weaker nonlinear central-cardiovascular and central-cardiorespiratory coupling pathways, and significantly stronger linear central influence on the vascular system, and on the other hand significantly stronger linear respiratory and cardiac influences on central activity in SZO compared to CON, and thus, providing better understanding of the interrelationship of central and autonomic regulatory mechanisms in schizophrenia might be useful as a biomarker of this disease.

## 1. Introduction

In the field of medical analysis, the interdisciplinary field of Network Physiology increasingly becomes the focus of interest in how insight may be gained into the coupling pathways of regulatory mechanisms in healthy and diseased states. Network Physiology aims to provide a better understanding of how the different integrated physiological (sub)-systems with their complex structures and regulatory mechanisms describe the global behavior and distinct physiologic functions at the organism level [1]. Thereby, analysis of structural, dynamical and regulatory mechanisms of the information transfer within these systems allows the characterization of healthy and diseased conditions [2]. The identification and quantification of complex dynamically acting systems with their different types of interrelationships is an continuing trail [3]. Recent advances in nonlinear dynamics, information theory and network theory lead to a new sophisticated knowledge about physiological structures and behaviors in health and disease states. Thereby, the connections between genetic and sub-cellular levels with intercellular information transfer among integrated organ systems and sub-systems are provided [2].

In short, schizophrenia represents a severe mental disorder, and is associated with higher cardiac mortality rates, and up to a 3-fold increased risk for cardiovascular disease (CVD) compared to the general population, independent of age groups [4,5,6]. In different studies, it was demonstrated that the regulation of the autonomic nervous system (ANS), is altered and impaired in schizophrenia, shown by analyzing heart rate variability (HRV) [7,8,9,10,11], respiratory variability (RESPV) [12,13,14,15,16,17], and cardiovascular- and cardiorespiratory couplings [14,15,18,19,20,21]. These different studies clearly hallmarked that for schizophrenia the variability of heart rate and respiration and the coupling between these systems are a disease inherent feature. Due to that the cardiovascular and cardiorespiratory system and their subsystems (ANS) are linked to the central nervous system (CNS) (sophisticated interplay between ANS and CNS). It can be assumed that this interplay based on a feedback-feedforward system supporting flexible and adaptive responses to environmental demands. Investigating the coupling between these ANS subsystem with their variability and brain activity might lead to a better understanding of pathophysiological regulatory processes within the central-autonomic network in those patients. In sum, it is assumed that a vagal withdrawal and an over activation of the sympathetic branches of the ANS are present in schizophrenia.

The complex interplay of the cardiovascular and cardiorespiratory system and their subsystems can be described as linear and nonlinear closed-loops with their feedforward and feedback mechanisms [22]. On important closed-loop is the arterial baroreflex control loop where alterations in blood pressure are detected by the baroreceptors leading to an adaptation of the heart rate. On the other hand, via the Windkessel function, heart rate alterations lead to changes in blood pressure [23]. The respiratory sinus arrhythmia (RSA) is one of the most important closed-loops within the cardiorespiratory system. RSA describes the rhythmic fluctuation of heart rate in regard to respiration commonly described as changes between inspiratory heart rate acceleration and expiratory heart rate deceleration under normal physiological conditions [24]. Two main regulatory mechanisms are present, the central information transfer towards respiration on vagal cardiac motoneurons, and the influence of respiration on intrathoracic pressure and stroke [24,25,26,27]. It is recognized that the respiration-RSA phase relationship is dependent, in part, nonlinearly on breathing frequency [28,29]. Thereby, RSA (or high-frequency HRV) is frequently employed as an index of cardiac vagal tone or even believed to be a direct measure of vagal tone [30]. It could be shown that RSA increases with increasing RMSSD (as a HRV measure of vagal tone in cardiac and respiratory system) [31]. HRV is determined mainly by parasympathetic regulation based on fast metabolism of acetylcholine (sympathetic nerve effect occurs with latency). HRV analysis in the frequency domain allows to characterize respiratory-related effects on the high-frequency band of the HRV (HF: 0.15–0.4 Hz) whereas low-frequency band (LF: 0.04–0.15 Hz) is influenced by contribution of sympathetic modulation mediated by baroreceptor activity. The respiratory-linked HRV indexed by high-frequency HRV (HF) is associated with several central regions such as prefrontal or cingulate cortex, and thus, RSA reflects the ‘brain-heart’ bidirectional information transfer. Ref. [32] RSA magnitude (HF) at rest and during stress could provide important information related to physiological and behavioral flexibility of the organism including emotional regulation (i.e., mental disorders) [32].

The complex interplay of the CNS and autonomic nervous system (ANS) with their large amount of subsystems (parasympathetic and sympathetic activity) is also known as the central-autonomic-network (CAN). It was shown, that the output of CAN is directly linked to ANS (heart rate) as well as that sensory information from peripheral end organs providing feedback to the CAN (i.e., baroreceptor reflex). The information transfer between the CNS and ANS is characterized as a feedback-feedforward system that responds to substantial demands of the body. The cerebral cortex in autonomic control of the cardiovascular system is gaining increased attention in medicine. Different cardiovascular control centers in the brainstem deal with different reflex mechanisms of cardiovascular adjustment (i.e., the cardiopulmonary reflex, the chemoreflex and the baroreflex) [33]. Here, neurons in the caudal and rostral ventrolateral medulla affecting efferent sympathetic reflexes, and contribute to the maintenance of heart rate and blood pressure via the intermediolateral cell column of the spinal cord. The two medullary areas, the nucleus ambiguous and the dorsal motor nucleus of the vagus nerve are preganglionic parasympathetic neurons mediating the efferent parasympathetic reflex mechanism [34,35]. The parasympathetic nervous system is responsible for “rest and digest” function, when you are sitting, resting and relaxing. It constricts the pupil, slows heart rate and contractility, contracts bronchial musculature and stimulates bronchial secretions, and enhances gut motility for digestion. The preganglionic neurons synapse onto postganglionic neurons in the parasympathetic ganglion that are located next to, or in, the effector end organs. The sympathetic nervous system predominates during “fight-or-flight” reactions and during exercise and thus prepares the body for stressful physical activity. Sympathetic nervous activity increases the flow of blood that is well-oxygenated and rich in nutrients to the tissues that need it, in particular, the working skeletal muscles. The preganglionic sympathetic neurons arise from the thoracic and lumbar regions of the spinal cord (segments T1 through L2) and are located about halfway between the CNS and the effector tissue [36]. The preganglionic neurons of both the sympathetic and parasympathetic divisions release the neurotransmitter acetylcholine. The postganglionic neurons of the parasympathetic system also release acetylcholine, whereas, the postganglionic sympathetic neurons release norepinephrine [37]. The cardiac or respiration-related activity (parasympathetic) is connected to preganglionic neurons. It was shown, that brain regions like the insula, thalamus, hypothalamus, amygdala, and the medial prefrontal cortex are involved in the autonomic regulation at rest and during cognitive or emotional stress conditions proven by functional brain imaging [38,39]. Beissner et al. [40], showed that largely divergent brain networks were associated with sympathetic and parasympathetic activity. The ventromedial prefrontal cortex (VMPFC), the perigenual anterior cingulate cortex (pACC), the dorsal anterior cingulate cortex (dACC), the posterior cingulate cortex (PCC), the insular cortices and amygdala seem to be the main cortical and subcortical areas involved in ANS regulation processes, that are created by a network of interactions related to task and autonomic division. Schizophrenia and other psychopathological conditions i.e., anxiety, depression and post-traumatic stress disorder are linked with prefrontal hypoactivity and a lack of inhibitory neural processes denoted by poor affective information processing and regulation [41].

For the quantitative analysis of the brain-heart (CNS-ANS) network coupling pathways and its integrated interacting subsystems as the cardiovascular and cardiorespiratory system several linear/nonlinear univariate and multivariate approaches are available. These approaches focus on characterizing the multivariate information transfer. These concepts [1,2,42,43] are applicable in the following domains: Entropy, Granger causality; nonlinear prediction; phase synchronization, symbolization, recurrence quantification analysis (RQA) and functional connectivity analysis techniques [18,44,45]. It has been demonstrated, that the information transfer between the cardiovascular- and cardiorespiratory system acts strongly nonlinear [46], and therefore linear approaches alone are not fully able to quantify physiological as well as pathophysiological regulatory processes.

This study aimed to characterize short-term instantaneous central-autonomic-network coupling pathways (top-to-bottom and bottom to top) by analyzing the interaction of heart rate, systolic blood pressure, respiration and central activity (EEG) in schizophrenic patients. Therefore, we applied causal linear and nonlinear multivariate coupling approaches (normalized short time partial directed coherence, multivariate transfer entropy) that are able to determine causal coupling strengths and directions within the CNS-ANS network. The coupling analyses are based on the indices mean and standard deviation of HRV, RESPV and systolic blood pressure. We believe that these findings are of importance for a full comprehension of (patho)physiological regulation processes and might allow improvement in treatment strategies in schizophrenic patients, and finally, possibly contribute to cardiac risk stratification strategies able to identify those patients at higher risk for cardiovascular disease. 

## 2. Materials and Methods 

### 2.1. Subjects

We enrolled 17 patients with paranoid schizophrenia (SZO; 2 females, 37.5 ± 10.4 years) in comparison to 17 healthy subjects (CON; 4 females, 37.7 ± 13.1 years). The diagnosis of schizophrenia was reached through an assessment of patient-specific signs and symptoms, as described in the Diagnostic and Statistical Manual of Mental Disorders, 4th edition (DSM-IV) [47]. The positive and negative syndrome scale was applied to quantify psychotic symptoms. Depot antipsychotic medication (77% being atypical neuroleptics [Seroquel, Risperdal, Olanzapin, Leponex, Seroquel, Zypadhera, Clozapin, Xeplion, Solian], and 23% being a mixture of antidepressant and atypical neuroleptics [Remergil, Zyprexa, Haloperidol, Seroquel, Flunxol, Leponex]) were used to treat SZO. Thoroughly performed interview and clinical investigations were performed for CON to exclude any potential psychiatric (DSM-IV) or other diseases, as well as to double-check for any interfering medication. The structured clinical interview and a personality inventory (Freiburger Persönlichkeitsinventar a factor-analytically and itemmetrically based method) were also applied to CON to detect personality traits and any disorders which might influence autonomic function [48]. To avoid hyperventilation all subjects were asked to relax and to breathe normally without any further breathing instructions. The written informed consent to a protocol approved by the local ethics committee of the Jena University Hospital was provided by all subjects. This study complies with the Declaration of Helsinki.

### 2.2. Data Recordings and Pre-Processing

**Figure 1:**
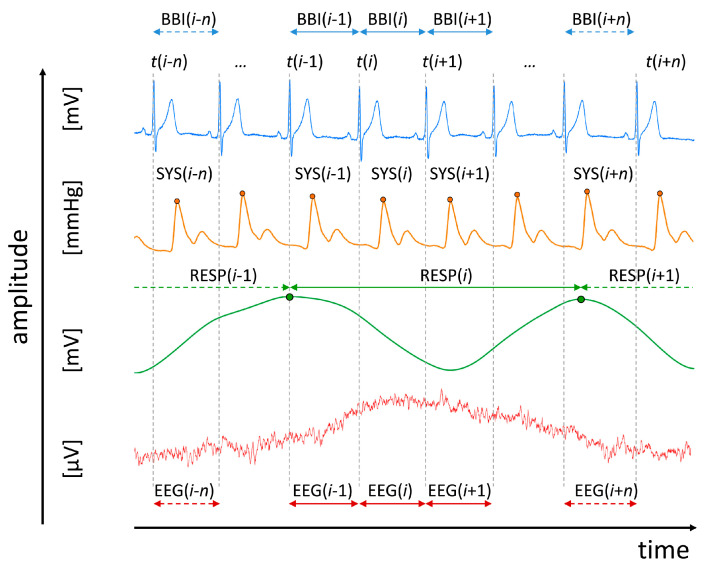
A visualization example of analyzed raw data records and their extracted time series. Raw data are, from top to bottom: ECG, non-invasive continuous blood pressure, synchronized calibrated respiratory inductive plethysmography signal (RESP), and electroencephalogram (EEG). RR(*i*) represents the beat-to-beat intervals; SYS(*i*) represents the maximum systolic blood pressure amplitude values over time in relation to the previous R-peak; RESP(*i*) represents the respiratory frequency as time intervals between consecutive breathing cycles, and EEG(*i*) specified the time intervals of the EEG raw data (electrode: Fp2) in relation to BBI(*i*). Within each EEG(*i*) the mean power P_EEG_(*i*) was calculated.

For all schizophrenic patients and healthy subjects, a 3-channel ECG (500 Hz), a synchronized non-invasive continuous blood pressure (200 Hz, volume-clamp photoplethysmographical technique, Portapres Model-2, TNO Biomedical Instrumentation, Den Haag, Netherlands) and calibrated respiratory inductive plethysmography signal (LifeShirt^®^, Vivometrics, Inc., Ventura, CA, USA), and a 64-channel EEG were recorded synchronously for 15 min. An extended 10–20-system using an electrode cap of 64 active Ag/AgCl electrodes was used to acquire the EEG (Brain Products, Gilching, Germany, AFZ: ground, FCZ: reference, *f* = 500 Hz). The impedance levels (<25 KΩ) were checked for each electrode before starting recordings. After a resting period of 10 min the recordings were started whereby, all subjects were asked to relax with closed eyes. ANS regulation was extracted from the following time series records (Figure 1):

- Heart rate (lead I) consisting of successive beat-to-beat intervals (BBI, tachogram, (msec));
- Maximum successive end-systolic blood pressure amplitude values over time in relation to the previous R-peak (SYS, systogram, (mmHg));
- Respiratory frequency (RESP, (sec)) as time intervals between consecutive breathing cycles.

With respect to each extracted BBI(*i*) (Figure 1) from the ECG raw data the related time intervals EEG(*i*) (msec) from EEG raw data were extracted). Within each EEG(*i*) within each corresponding BBI(*i*), with *i* (*i* = 1:*R* − 1) as the successive number of R-peaks (*R*), the mean power P_EEG_(*i*) (µV^2^) was derived representing time series of EEG activity [20].

(1) $$P_{\mathrm{EEG}}\left[i\right]=\frac{1}{T}\overset{t\left(i+1\right)\times fs}{\underset{j=t\left(i\right)\times fs}{\sum }}{\left|\mathrm{EEG}\left[j\right]\right|}^{2}$$

with *T* representing the number of samples within BBI(*i*) or EEG(*i*), respectively; *t*(*i*) represents the current point in time of BBI(*i*) and *fs* represents the sampling frequency.

EEG recordings (pre-processing) were band-pass filtered (0.05–60 Hz, Butterworth filter, order = 3) to eliminate slow drifts coming from slow body movements or sweating, and to exclude noise resulting from higher frequency contents. For the quantitative EEG analysis EEG time series were visually inspected and automatically classified as artefact-free applying the Brain Products Software Analyzer 2.0 to get artefact-free time series [49,50,51].

All extracted time series were adaptive filtered (adaptive variance estimation algorithm [52]) to exclude and interpolate ventricular premature events and/or artefacts to obtain normal-to-normal beat time series (NN). For coupling analysis all time series (BBI, SYS, RESP, and P_EEG_) were synchronized and resampled with a linear interpolation algorithm (2 Hz).

**Figure 2:**
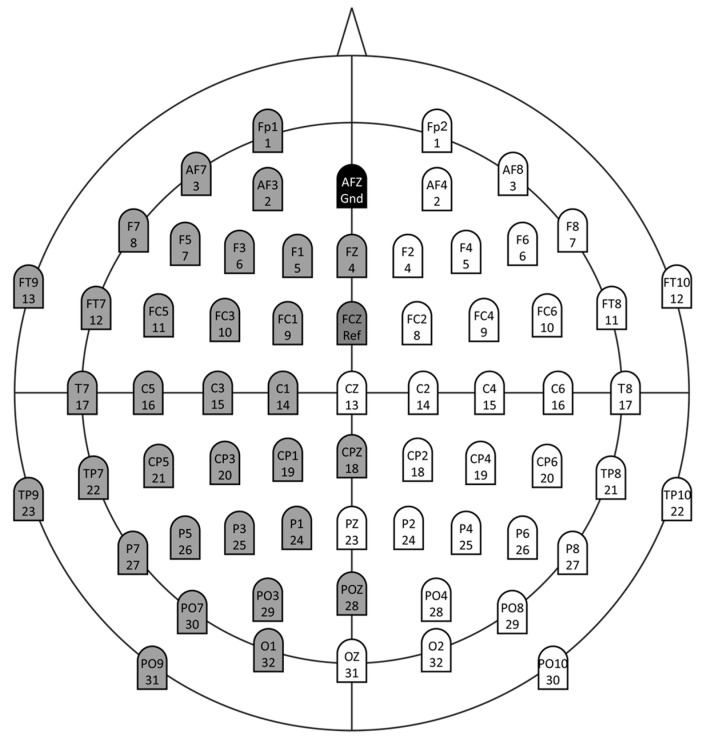
The extended 10–20 EEG system (actiCAP, Brain Products). (Grey marked channels belong to the left hemisphere and white marked channels belong to the right hemisphere; AFZ = ground (black); FCZ = reference (dark grey)).

The frontal lobe with the related EEG electrodes (AF3, AF4, AF7, AF8, Fp1, Fp2, F1, F2, F3, F4, F5, F6, F7, F8, Fz) were analyzed (central-cardiovascular couplings, central-cardiorespiratory couplings) (Figure 2).

### 2.3. Standard Indices from Electroencephalogram, Heart Rate-, Blood Pressure- and Respiratory Variability in the Frequency-and Time Domains

From the EEG raw data the power spectral density (PSD) function (window length = 5 sec, overlap = 50%) applying an autoregressive model (Welch’s method) [49] was used to estimate the mean power (P; 0.5–60 Hz) to quantify the electroencephalogram.

Standard indices from time domain [53] for the quantitative analysis of heart rate variability (HRV), blood pressure variability (BPV), and respiratory variability (RESPV) were derived as:

- meanNN: the mean value of the NN intervals of BBI (msec), of systolic (SYS) blood pressure (mmHg) values, and RESP (sec) as respiratory cycle length;
- sdNN: the standard deviation of the NN intervals of BBI (msec), of systolic (SYS) blood pressure (mmHg) values, and RESP (sec);
- BF: the breathing frequency characterizing the number of breaths per minute (1/min).

### 2.4. Central-Cardiovascular and Central-Cardiorespiratory Coupling Analyses

For the quantification of linear and nonlinear central-cardiovascular and central-cardiorespiratory couplings different approaches can be used [43]. In this investigation, we analyzed the information transfer between BBI, SYS, RESP and P_EEG_ time series with the linear causality approach of the normalized short-time partial directed coherence (NSTPDC) [54], and the nonlinear multivariate Transfer Entropy (MuTE) [55] approach.

#### 2.4.1. Normalized Short-Time Partial Directed Coherence

The normalized short-time partial directed coherence (NSTPDC) represents an extension of the classical partial directed coherence (PDC) [56] that is able to detect both direct and indirect causal couplings within multivariate time series. The time-variant partial directed coherence approach (tvPDC, $\pi_{xy}\left(f,n\right)$) is the fundamental basis of the NSTPDC quantifying partial correlative short-time couplings of non-stationary signals at the frequency *f* within windows (*n* the number of windows) [57]. The basis of the NSTPDC represents an *m*-dimensional autoregressive (AR) model with the order *p* and allows determining linear Granger causality in the frequency domain. The optimal model order *p*_opt_ of the AR model and its coefficients the stepwise least squares algorithm [58] and the Schwarz’s Bayesian Criterion (SBC) were applied [59]. To estimate the coupling direction between two time series, *x* and *y* (e.g., BBI and P_EEG_: with *x*_BBI_ and *y*_PEEG_) with the covariate *z* (e.g., SYS with *z*_SYS_) NSTPDC works in principle like that a coupling factor (CF) was derived by dividing the mean value $\pi_{xy}\left(f,n\right)$ by the mean value of $\pi_{yx}\left(f,n\right)$.

(2) $$\mathrm{CF}=\frac{\frac{1}{n}\sum \pi_{xy}\left(f,n\right)}{\frac{1}{n}\sum \pi_{yx}\left(f,n\right)},\bar{a}=\frac{1}{n}\sum \pi_{xy}\left(f,n\right),\bar{b}=\frac{1}{n}\sum \pi_{yx}\left(f,n\right)$$

The results of CF were normalized, producing the normalized factor (NF) that characterizes the coupling direction.

(3) $$\max\left(\bar{a},\bar{b}\right)\;\mathrm{NF}=\left\{\begin{array}{c} 2,if\left(\max=\bar{a}\wedge \frac{\bar{a}}{\bar{b}}>5\right)\\ 1,if\left(\max=\bar{a}\wedge 2<\frac{\bar{a}}{\bar{b}}\leq 5\right)\\ 0,if\left(\max=\bar{a}\wedge 0\leq \frac{\bar{a}}{\bar{b}}\leq 2\right) \end{array}\right\}\mathrm{and}\mathrm{NF}=\left\{-\begin{array}{c} -2,if\left(\max=\bar{b}\wedge \frac{\bar{b}}{\bar{a}}>5\right)\\ 1,if\left(\max=\bar{b}\wedge 2<\frac{\bar{b}}{\bar{a}}\leq 5\right)\\ 0,if\left(\max=\bar{b}\wedge 0\leq \frac{\bar{b}}{\bar{a}}\leq 2\right) \end{array}\right\}$$

Thereby, NF (NF = {−2, −1, 0, 1, 2}) determinates the causal coupling direction between the set of time series (*x*_BBI_ and *y*_PEEG_) as a function of frequency *f*. 

*Coupling direction:*

- NF = {−2 | 2} (where −2 denotes *y*_PEEG_ as the driver): *Strong unidirectional* coupling;
- NF = {−1.5 to −2} or NF = {1.5 to 2}: *Weak unidirectional* coupling;
- NF = {−1 | 1} (−1 denotes *y*_PEEG_ as the driver): *Strong bidirectional* coupling;
- NF = {−0.5 to −1} or NF = {0.5 to 1}: *Weak bidirectional* coupling;
- NF = 0: *Equal influence in both directions and/or no coupling* in respect to the coupling strengths (If both area indices reveal equal values larger than zero an equal influence in both directions is present, if both area indices reveal equal values but are zero no coupling is present).

*Coupling strength:*

The coupling strength can be determined by the area that is formed by CF and estimated in each window (*f* = 0−2 Hz). For two time series, e.g., *x*_BBI_ and *y*_PEEG_ with covariate (*z*_SYS_) these areas are: $A_{\mathrm{BBI}\to P_{\mathrm{EEG}}\left(\mathrm{SYS}\right)}$ and $A_{P_{\mathrm{EEG}}\to \mathrm{BBI}\left(\mathrm{SYS}\right)}$ [a.u.]. These area values range between 0 and 1 [0,1] (1 indicates that all causal information from *x* is transferred (→) towards *y* ($A_{x\to y\left(z\right)}$ = 1)). A window (Hamming) of length 120 samples, and shifting the window by 30 samples per each iteration step was used. With respect to scale-invariance all time series BBI, SYS, RESP and P_EEG_ were normalized to zero mean and unit variance [19]. 

#### 2.4.2. Multivariate Transfer Entropy

In 2000, Schreiber [60] introduced the Transfer Entropy (TE) to detect the information transfer between joint processes. TE is able to detect directional interactions between processes (driver-response-relationship) and asymmetries in these interactions. TE has the big advantage that it is a “model-free” approach [43] making TE very sensitive to any types of dynamical information transfer. Montalto et al. [55] provided a MATLAB toolbox with different entropy estimators (binning, nearest neighbor, linear) which constitutes the basis for TE in multivariate systems, the Multivariate Transfer Entropy (MuTE). MuTE extended the classical TE to the case of multiple interacting processes and discovers purely nonlinear interactions with a range of interaction delays. Hereby, the nonlinear causal coupling strength originating from time series *X* directed (→) towards time series *Y* conditioned by time series *Z* is expressed as:

MuTE*_X_*_→*Y*(*Z*)_ (4)

In this study, we used the nearest neighbor estimator and non-uniform embedding (NN NUE) technique for the nonlinear characterization of the multivariate information transfer [55] within the CAN, since it was shown, that high sensitivity and specificity for a linear and nonlinear systems can be archived when using NN NUE.

#### 2.4.3. Surrogate Data 

When applying nonlinear analysis approaches, it must be taken into account that the linear properties of the signals, like e.g., autocorrelation or spectral features, are likely to affect the measure. To demonstrate the statistical validity of the obtained network pathways between CON and SZO, surrogate tests were performed to determine a threshold for statistical significance for the obtained results [61,62]. The idea behind this technique is to apply the nonlinear method in question to independent time series that are the same or as close as possible to the statistical properties of the original time signals, while randomizing the expressions of the nonlinear property to be measured. This procedure makes it possible to define a threshold below which any result is considered to be false. In practice, when deriving couplings even from very weakly coupled (or completely decoupled) systems, the methods always capture some nonzero values of the apparent coupling strength. Surrogate testing can then be used to establish the “zero-level” of apparent coupling corresponding to uncoupled signals [63]. Therefore, this approach was applied for each subject (CON, SZO) and each original time series (BBI, SYS, RESP and P_EEG_) 15 independent surrogates were derived by randomly permutation of the temporal structure of the original samples to remove any temporal relationship for the new derived surrogate time series (CONsu, SZOsu). This technique preserves the linear structures of the signals, but changes the nonlinear properties.

A statistical significance thresholds *t*_su_ was defined, beneath which any coupling result derived from the original time series is considered spurious. This threshold was calculated independently for each subject were then set as the mean + 2*SD of the resultant distributions. As a result, we tested the couplings from the original time series by comparison with the significance threshold *t*_su_. Hence, if couplings were higher (original time series) than *t*_su_, and no significant differences between CONsu and SZOsu existed, then the null hypothesis was rejected, and significant couplings within the original time series were present. The non-parametric paired Mann-Whitney *U*-test was used to determine the significance of differences between the CONsu and SZOsu distributions. The test rejected the null hypothesis at the 0.22% significance level (Bonferroni-Holm).

### 2.5. Statistics

Significant differences between CON and SZO were estimated applying the nonparametric exact two-tailed Mann-Whitney *U*-Test (SPPS 21.0). The significance was considered at * *p* < 0.05, ** *p* < 0.01, *** *p* < 0.0022 (Bonferroni-Holm adjustment, *n* = 23 indices), and # not confirmed by surrogate analysis. Results in the following tables are presented as mean ± SD.

**Figure 3:**
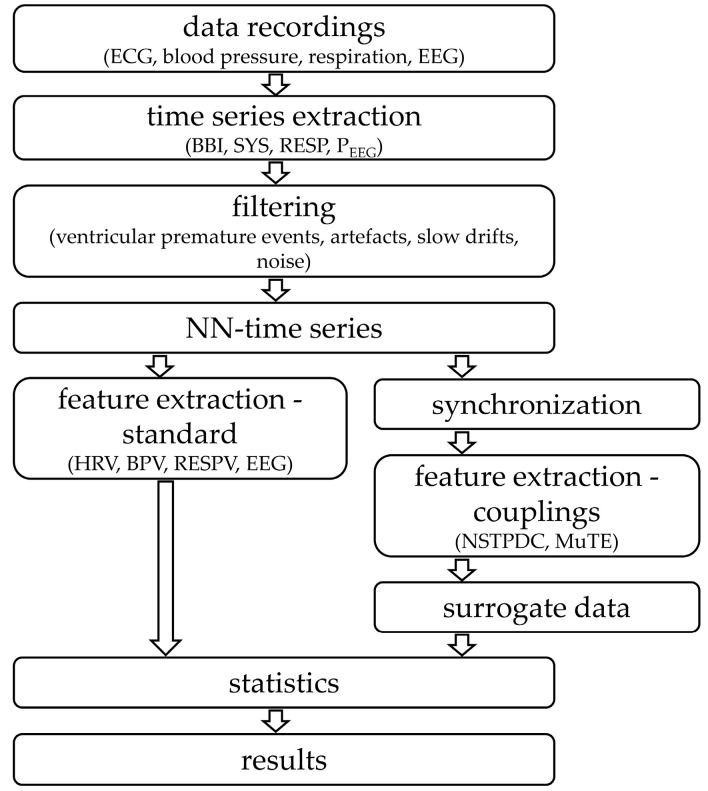
Flowchart of all performed analyses steps. (BBI represents the beat-to-beat intervals; SYS represents the maximum systolic blood pressure amplitude values over time in relation to the previous R-peak; RESP represents the respiratory frequency as time intervals between consecutive breathing cycles, P_EEG_ specified the mean power in the time intervals of the EEG raw data in relation to each BBI, NN: Normal-to-normal beat interval, HRV: Heart rate variability, BPV: Blood pressure variability, RESPV: Respiratory variability, NSTPDC: Normalized short-time partial directed coherence, and MuTE: Multivariate Transfer Entropy).

An overview of all performed analyses steps are presented in Figure 3.

## 3. Results

### 3.1. Standard Indices from Electroencephalogram, Heart Rate Variability, Blood Pressure Variability and Respiratory Variability in the Frequency-and Time Domains

Significant differences between CON and SZO were demonstrated in the time domain of HRV analysis (meanNN_BBI_, sdNN_BBI_) (Table 1). SZO showed an increased heart rate (reduced meanNN_BBI_) accompanied with reduced (↓) variability (sdNN_BBI_) compared to CON. Blood pressure variability and respiratory variability analysis did not show any significant difference between both groups. Analysis of the EEG (PSD) revealed significant differences between CON and SZO, whereby, SZO presented a significant decrease in the mean power P of PSD (Table 1).

### 3.2. Central-Cardiovascular Coupling-Linear

#### 3.2.1. Cardiovascular Coupling

NSTPDC results demonstrated a significant different coupling direction (NF) in SZO (NF~−0.5) compared to CON, pointing to a decreased bidirectional coupling in the direction from SYS→BBI (SYS is the driver). The coupling strength from SYS→BBI ($A_{\mathrm{SYS}\to \mathrm{BBI}\left(P_{\mathrm{EEG}}\right)}$), known as baroreflex loop, was significantly reduced in SZO (↓) in comparison to CON (Table 2).

#### 3.2.2. Central-Cardiac Coupling

If central activity (P_EEG_) was coupled with cardiac activity (BBI) the NF value showed a significant different behavior between both groups. For SZO (NF ~ −0.8) the coupling direction was characterized as a bidirectional one from P_EEG_→BBI (P_EEG_ is the driver) (Table 2).

#### 3.2.3. Central-Vascular Coupling

The coupling between the vascular system (SYS) and the central system (P_EEG_) a highly significantly different NF value was present for both groups. The coupling direction were characterized as, that CON (NF~0) pointing to an equal information transfer in both directions, and SZO (NF ~ −0.7) indicating a bidirectional one from P_EEG_→SYS (driver P_EEG_). These results were supported by $A_{\mathrm{SYS}\to P_{\mathrm{EEG}}\left(\mathrm{BBI}\right)}$ and $A_{P_{\mathrm{EEG}}\to \mathrm{SYS}\left(\mathrm{BBI}\right)}$ for CON demonstrating similar values for the area indices for both coupling directions. $A_{\mathrm{SYS}\to P_{\mathrm{EEG}}\left(\mathrm{BBI}\right)}$ and $A_{P_{\mathrm{EEG}}\to \mathrm{SYS}\left(\mathrm{BBI}\right)}$ were highly significantly different between SZO and CON. The coupling strength was significantly reduced in SZO when SYS influenced P_EEG_ (SYS→P_EEG_) compared to CON. If P_EEG_ influences SYS (P_EEG_→SYS) a significant increase in the coupling strength were present for SZO compared to CON (Table 2).

### 3.3. Central-Cardiovascular Coupling-Nonlinear

#### 3.3.1. Cardiovascular Coupling

MuTE revealed a highly significant different coupling strength between SZO and CON. Considering the baroreflex loop when SYS influenced BBI (MuTE_SYS_→_BBI(PEEG)_), in contrast to the linear results, the coupling strength was significantly increased in SZO compared to CON, and might point to a stronger nonlinear causal information transfer of SYS on BBI. When BBI influenced SYS (MuTE_BBI_→_SYS(PEEG)_), the coupling strength was highly significantly different between SZO (↓) and CON (Table 3).

#### 3.3.2. Central-Cardiac Coupling

The nonlinear causal information transfer from cardiac system to the central system (BBI→P_EEG_) as well as from central system to cardiac system (P_EEG_→BBI) were highly significantly reduced in SZO in comparison to CON, and were nearly equally strong pronounced in SZO (Table 3).

#### 3.3.3. Central-Vascular Coupling

The nonlinear central-vascular couplings from the vascular system to the central system (SYS→P_EEG_) as well as from central system to vascular system (P_EEG_→SYS) were significantly reduced in SZO compared to CON, and were nearly equally strong pronounced in both groups (Table 3).

### 3.4. Central-Cardiorespiratory Coupling-Linear

#### 3.4.1. Cardiorespiratory Coupling

NSTPDC revealed a significant different coupling direction between SZO and CON. SZO (NF~−1.5) showed to a bidirectional information transfer (RESP→BBI) (RESP is the driver). The coupling strength for the information transfer from the cardiac system to the respiratory system ($A_{\mathrm{BBI}\to \mathrm{RESP}\left(P_{\mathrm{EEG}}\right)}$) was highly significantly decreased for SZO compared to CON. For the RSA loop, when the respiratory system (RESP) transfers information towards the cardiac system (BBI) the coupling strengths were increased for both groups, but not significantly different (Table 4).

#### 3.4.2. Central-Cardiac Coupling

For central-cardiac coupling analysis (BBI-P_EEG_) all indices showed significant difference between both groups. For the coupling direction, SZO (NF ~ −0.1) showed an equal influence in both directions and/or no coupling (P_EEG_↔BBI).

The coupling strength ($A_{\mathrm{BBI}\to P_{\mathrm{EEG}}\left(\mathrm{RESP}\right)}$) for the information transfer from cardiac system to the central system (BBI→P_EEG_) showed highly significant differences between SZO and CON. Here, SZO presented a stronger coupling from BBI→P_EEG_ compared to CON. In the case of, when the information transfer is from central system directed towards the cardiac system (P_EEG_→BBI) the coupling strength ($A_{P_{\mathrm{EEG}}\to \mathrm{BBI}\left(\mathrm{RESP}\right)}$) were significantly increased in SZO, and vice versa. For SZO the strength of the information transfer from the central system (P_EEG_) towards the cardiac system (BBI) was increased, and vice versa, whereas for CON it was much more pronounced from the central system towards the cardiac one (P_EEG_↔BBI), and less pronounced and vice versa (Table 4).

#### 3.4.3. Central-Respiratory Coupling

The coupling direction (central-respiratory) revealed highly significantly differences between SZO and CON. SZO (NF ~1.3) and CON (NF ~1.0) presented both a strong bidirectional one (RESP→P_EEG_) (RESP is the driver) and were confirmed by highly significantly different coupling strengths ($A_{\mathrm{RESP}\to P_{\mathrm{EEG}}\left(\mathrm{BBI}\right)}$, $A_{P_{\mathrm{EEG}}\to \mathrm{RESP}\left(\mathrm{BBI}\right)}$) between both groups. The coupling strength for the information transfer from the respiratory system to the central system (RESP→P_EEG_) was significantly increased in SZO compared to CON. For the information transfer from the central system to the respiratory system (P_EEG_→RESP) the coupling strengths indicated a significant reduction in SZO vs. CON. The coupling strength from the ANS (RESP) towards CNS (P_EEG_) was more pronounced than vice versa, whereby, for CON it was expressed in an opposite way (Table 4).

### 3.5. Central-Cardiorespiratory Coupling-Nonlinear

#### 3.5.1. Cardiorespiratory Coupling

Nonlinear cardiorespiratory coupling analyses revealed that the coupling strengths from BBI to RESP and from RESP to BBI (RSA loop) were significantly decreased in SZO in comparison to CON (Table 5).

#### 3.5.2. Central-Cardiac Coupling

Causal nonlinear central-cardiac coupling was only significantly different between SZO and CON in the case when BBI influenced P_EEG_ (Mu_TEBBI_→_PEEG(RESP)_). The nonlinear influences from BBI to P_EEG_ as well as from P_EEG_ to BBI were nearly comparable in both directions, and slightly more pronounced in CON than SZO (Table 5).

#### 3.5.3. Central-Respiratory Coupling

Central-Respiratory coupling analyses revealed a similar behavior as shown for central-cardiac coupling analyses. High significant differences were found for both cases, when RESP influences P_EEG_ (MuTE_RESP_→_PEEG(BBI)_) as well as when P_EEG_ influences RESP (MuTE_PEEG_→_RESP(BBI)_). The nonlinear information transfer from respiratory activity towards central activity and from P_EEG_ to RESP were nearly comparable in both directions and more pronounced in CON than SZO (Table 5).

## 4. Discussion

**Figure 4:**
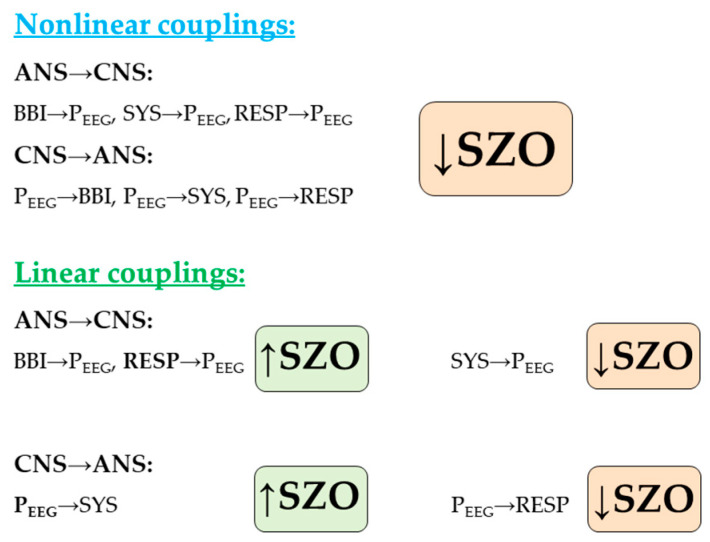
Summary of linear and nonlinear coupling results in schizophrenia (SZO) in comparison to healthy subjects (CON). (BBI: beat-to-beat intervals; SYS: End-systolic blood pressure amplitude values over time; RESP: Respiratory frequency; P_EEG_: The mean power in the BBI-related EEG intervals).

In our study, we demonstrated highly significant altered central-autonomic couplings in patients with paranoid schizophrenia in comparison to healthy subjects. For SZO these couplings were characterized as bidirectional ones, with more pronounced central driving towards SYS (P_EEG_→SYS) than SYS (SYS→P_EEG_) towards CNS, and stronger respiratory driving towards P_EEG_ (RESP→P_EEG_) and cardiac driving towards P_EEG_ (BBI→P_EEG_) than vice versa, compared to CON (Figure 4).

Central-cardiovascular interactions revealed that a stronger linear information flow from central activity in the direction of blood pressure regulation (SYS) than in the direction of BBI in SZO compared to CON. In particular, in SZO, the central-cardiac information transfer is more nonlinearly defined and significantly bidirectionally decreased. This suggests for SZO, that the linear central-vascular regulation closed-loop (baroreflex loop) purposefully maintains the blood pressure adaptation and is more aligned than the nonlinear part of this regulation closed-loop. Especially, for SZO, within this closed-loop it is obvious that the central regulatory processes (P_EEG_) are more directed towards the vascular system (SYS) than in the opposite direction. The altered central-cardiorespiratory couplings in SZO are characterized by a weaker nonlinear central information transfer (P_EEG_→BBI) in the direction of the cardiac system, and stronger linear respiratory and cardiac information flows in the direction of the central system (RESP→P_EEG_, BBI→P_EEG_) compared to CON.

This is in accordance to other findings that the coupling between the central and autonomic nervous systems is driven by quite complex regulatory mechanisms where, in general, the CNS commands and the ANS reflexes [64]. Recently, it was well demonstrated that beyond a dysfunction of connectivity among different brain areas in schizophrenia, there is also an abnormal asymmetry of functional connectivity and a failure of the left-hemisphere dominance compared with healthy subjects. Moreover, an overall generally attenuated asymmetry of functional connectivity that increases with the duration of the disease and correlates with psychotic symptoms in SZO was found [65,66]. This abnormal asymmetry of connectivity may be related to a dysfunctional inter-hemispheric communication [65]. This asymmetry is characterized by failure of the left hemisphere dominance (parasympathetic tone) in SZO and is located in several frontal regions and the hippocampus. This asymmetry of functional connectivity in schizophrenia, suggests that this aspect may represent a neurophysiological feature that is unique to this disorder [65]. In respect to cardiovascular activity extensive research has been conducted on the influence of the frontal, temporal, and parietal regions on heart rate and blood pressure regulation, where, the two hemispheres seem to possess contrasting roles in regulating changes in heart rate and blood pressure. Different studies consistently found a relationship between changes in heart rate and blood pressure and measures of cerebral activity at these locations (temporal and posterior regions), such as electroencephalography (EEG) [40,67]. Significant correlations between resting heart rate and frontal lobe lateral asymmetry as well as frontal–parietal asymmetry was found supporting the relative differential associations of the left and right frontal and parietal lobes and cardiovascular activity [67]. Due to this asymmetry the relative right frontal activation will generate increased inhibition of the right posterior region as well as decreased left frontal lobe activation, resulting in increased left posterior (parasympathetic) activity. Conversely, relative left frontal lobe activation will cause increased inhibition of the left posterior region as well as decreased right frontal lobe activity, resulting in increased right posterior (sympathetic) activity. There is evidence for an inhibitory role of the frontal lobes; stimulation of the medial prefrontal regions generates bradycardia and depressor responses and inhibition of conditioned increases in heart rate and blood pressure. [67] Resting heart rate was associated with lateral asymmetry across the frontal and parietal lobes, resting systolic and diastolic blood pressure were related to lateral asymmetry across the temporal and parietal lobes [68].

What this means for SZO can only be speculated with this study. In general, the CAN represents a dynamic system, with neural structures involved in affective and autonomic regulation, especially cardiovascular activity [69,70]. The CAN controls preganglionic sympathetic and parasympathetic, neuroendocrine, respiratory, and sphincter motoneurons and is characterized by reciprocal interconnections, parallel organization, state-dependent activity, and neurochemical complexity [71]. Thereby, parasympathetic activation decreases the firing rate of pacemaker cells and heart rate, while sympathetic activity results in an increase of heart rate and firing rate of the pacemaker cells in the heart sinoatrial node [72,73]. For SZO, it can be assumed, that through the failure of the left-hemisphere activity in the frontal lobe, the parasympathetic tone is inhibited and thus the sympathetic tone is overactive resulting in increased heart rate and blood pressure values. As a consequence, within the CAN, the central activity (P_EEG_) will be increased to counteract these phenomena in the periphery (ANS) to decrease at least the blood pressure. 

The closed-loop (feedback-loop) of central-cardiorespiratory regulation seems to be focused on adjusting this increased cardiac activity (heart rate) via the sinus node rather than on regulating respiration. However, whether central chemoreceptors regulate the cardiovagal outflow independently of the respiratory system is an open question [74]. The respiratory network receives peripheral chemosensory and mechanosensory inputs and modulatory inputs from the other parts of the brain. These inputs are essential for adaptive changes in the respiratory motor output, ensuring appropriate ventilation of the lungs in variable environmental and physiological conditions [75]. Lung ventilation, cardiac output, and blood pressure are highly labile physiological sates that are continually adjusted by the CNS to match the metabolic requirements of specific behaviors [74]. It seems to be that in SZO maintaining the oxygen supply takes priority expressed by the stronger feedback from RESP towards P_EEG_. This feedback-loop from RESP towards central activity is strongly dominated by respiratory activity. This could be originate by reflexes from muscle mechano- and metabotropic receptors cooperate to activate breathing to a degree roughly commensurate with the rise in whole body metabolism and oxygenation. It seems that these cardiorespiratory responses were caused by an initial fast increase in cardiovascular and ventilatory flow parameters that are brought about by neurally mediated muscle mechanoreceptor feedback reflexes and a feedforward ‘central motor command’. The combination of these two neural mechanisms will also increase the blood pressure operating point. Thus, the fine control of the matching of cardiac output to ventilation may occur by means of a feedforward ventilatory control of cardiac origin [76].

For medicated schizophrenic patients it was clearly demonstrated in different studies that these patients revealed an ANS dysfunction expressed by a decreased HRV (sdNN_BBI_↓, meanNN_BBI_↓). The strongest anticholinergic side effects of cholinergic or adrenergic receptors on ANS regulation are related to antipsychotics as clozapine, quetiapine and amisulpride, and rather than olanzapine [77]. Thus, the altered HRV and central-autonomic coupling in SZO cannot only be associated with antipsychotic medications. It was shown by Agelink et al. [78], that different atypical antipsychotics have different effects ANS regulation, whereby, only amisulpride did not significantly activate cholinergic or adrenergic receptors and thus, did not affect HRV. The antipsychotic olanzapine appears to be associated with a decrease in vagal cardiac tone. [79], and only restricted to cardiac modulation [80]. The current SZO patients were treated with atypical neuroleptics and therefore a side effect of these drugs cannot be completely excluded on HRV and coupling results. An impaired cardiac regulation, as one major contributor to the altered ANS regulation expressed by increased basic heart rates in the psychotic state as well as in an unmedicated state, seems to be obviously present in schizophrenic patients demonstrated by different studies. Respiratory variability analysis showed only trends between SZO and CON, in contrast to other findings dealing with unmedicated patients [12,15,16]. These studies demonstrated significantly reduced inspiration and expiration times and increased breathing rates for schizophrenia as a unique feature during the psychotic state. Due to that the neurobiological mechanism of antipsychotic action is associated with D2 receptor antagonism as a pharmacodynamics property experts hypothesize that schizophrenia is associated with a dysregulation of dopaminergic circuits with excessive dopaminergic activity in the mesolimbic pathway as well as restricted dopaminergic activity in the mesocortical pathway [81,82]. Due to antipsychotics being anti-dopaminergic they probably have dopamine stimulating effect on respiration possibly leading to the reduced respiratory variability. Other studies support dopaminergic hypofunction in the cerebral cortex and hyperfunction in subcortical brain regions in SZO, as well as those typical antipsychotics (here dopamine) inhibit mitochondrial respiration [83,84]. The final respiratory output involves a complex interaction between the brainstem and higher centers, including the limbic system and cortical structures. Respiration is primarily regulated for metabolic and homeostatic purposes in the brainstem and also changes in response to changes in emotions, such as sadness, happiness, anxiety or fear [85]. In addition, it could be shown that anxiety is associated with changed respiratory patterns and respiratory frequency [86,87]. These studies showed that an increase in the respiratory frequency is not related to metabolic factors and is consistent with a mechanism involving the limbic system modulating respiratory drive [88]. The found alterations in respiration (trend) can be explained by a dysregulation of arousal, as suggested in paranoid schizophrenia in amygdalae prefrontal circuits and might contribute to the correlation of psychopathology and breathing alterations [12]. Schizophrenic patients who were taking clozapine and olanzapine in comparison to patients who were antipsychotic naive might have a compensatory mechanism in the neurobiological substrate, whereby, alterations in the anterior cingulate, the medial frontal cortex and a decline of fast frequency activities in the occipital cortex were related to clozapine and olanzapine (in this study most patients were treated with these atypical antipsychotic) [89]. 

The results of EEG frequency analyses demonstrated a decrease of central activation (power) in SZO in comparison to CON. It was proven [90], in general, that atypical antipsychotics like clozapine increase the central activation (power) in the δ and θ bands, especially in frontal brain regions, and thus, also directly affect, P_EEG_, The effects of placebo and antipsychotics (haloperidol, chlorpromazine, clozapine) were investigated by Small et al. [91], who demonstrated that clozapine and chlorpromazine are associated with increased frontal δ central activity. In addition, the effect of antipsychotic treatment (clozapine) on clinical scores was demonstrated by significantly reduced positive and negative symptoms and global psychopathology [92]. To the best of our knowledge, there are no comparative studies on the effects of antipsychotic treatment on central activity, so it is very difficult to assess its efficacy.

Linear cardiovascular coupling results showed a decreased bidirectional coupling strength in the direction from SYS→BBI in SZO indicating an inhibited baroreflex control loop, whereas the nonlinear part showed contrary results. We could show for SZO compared to CON, that the vascular system (SYS) is not affected by antipsychotic treatment leading to the assumption, that the inhibited baroreflex-loop is a result of an impaired cardiac modulation instead of an impairment of the blood pressure regulation circuit. In general, the arterial baroreflex is inhibited under stressful conditions [93,94,95] (as it is assumed for SZO). In stressful conditions, when the blood pressure increases, the baroreflex reduces sympathetic outflow and increases parasympathetic tone, which protects the heart, e.g., against arrhythmias. Both blood pressure buffering and cardioprotection are major effects of the arterial baroreflex [93]. Facilitation of stress favors restoration of energy exhausted during a stressful phase in which the subject reacts actively to changing environment. Thereby, brain regions which are related to central baroreflex regulation mechanisms and elicit facilitation of stress are the medial prefrontal cortex, the preoptic/anterior hypothalamus, the ventrolateral part of the periaqueductal grey matter, and the nucleus raphe magnus [94]. 

The stronger pronounced central information flow in the direction of vascular regulation (SYS) leads to the assumption that the central-cardiac information flow is probably restricted by the generally impaired cardiac regulation in schizophrenia independently of medication [7,19]. Lesions in the CNS (frontal and temporal lobes), as a part of the CAN, can lead to profound changes in heart regulation and even to potentially fatal cardiac arrhythmias or sudden cardiac death (cardiovascular dysfunctions) [96]. There exists a relationship between increasing magnitude of cerebral activation within the frontal and temporal lobes (regions are involved in the regulation of cardiovascular functioning) and changes in heart rate and blood pressure. That’s why our investigation was focused on EEG electrodes in these regions. Finally there is a well-known asymmetry between left and right hemisphere with failure of left side dominance that could influence the final coupling results. Thereby, increasing levels of cerebral activation within the left hemisphere would be associated with increasing parasympathetic tone and increasing levels within the right hemisphere with sympathetic tone [96]. However, it was stated that the two cerebral hemispheres act together to promote changes in cardiovascular functioning [97]. Other studies [67,98] showed that the two hemispheres are in a reciprocally balanced condition, with each hemisphere opposing and complementing the other one in respect to parasympathetic and sympathetic modulation of the cardiovascular function.

Peripheral end organs such as the heart (HRV) forward sensory information to the CAN and are directly linked, and thus can be used as a good qualitative characteristic of the central-peripheral neuronal feedback-loop [69]. Dysfunctions within the CNS and their connection to stronger pronounced dysregulation of cardiovascular regulation, characterized by cardiac- and vascular dysregulation expressed through increased abnormal top down modulation (brain to heart), might a reason for the increased risk of sudden cardiac death in SZO [96]. 

The linear coupling strength of the central-vascular axis (SYS→P_EEG_) was significantly decreased and oppositely aligned to the information flow from central activity towards ANS (P_EEG_→SYS) in SZO compared to CON. This axis was bidirectionally directed with a stronger central driving mechanism (P_EEG_→SYS) in SZO, whereas, CON showed equally directed information flows (P_EEG_↔SYS). This leads to the assumption that maintaining blood pressure as well as heart rate regulation takes on a greater importance for SZO expressed through increased top down regulation pathways, as for CON. In sum for SZO it is evident, that the linear central driving is more pronounced in the direction of autonomic activity (SYS) than in CON, and that the nonlinear information transfer within the central-vascular- and central-cardiac systems are reduced.

The direction (NF ~ −1.5) of the linear information flow within the cardiorespiratory system was bidirectionally pronounced and with the respiration as the driving part in the direction towards cardiac activity (RESP→BBI) in SZO compared to CON (NF ~ −1.6), who revealed a slightly better RSA-loop. The linear information flow BBI→RESP (significant) within the cardiorespiratory system is suggested as a biomarker complementing RSA as a reciprocal component of cardiorespiratory interaction [99]. Dick et al. [99] believe that this mutual interaction in the function of gas exchange between the respiratory and autonomic system is characterized in a way that the ANS transfers information to the respiratory system in generating breathing pattern is beat-to-beat, whereas the well-known information flow from respiration in the direction to ANS (RSA-loop) is pronounced breath-to-breath. In healthy subjects, it was shown that the degree of sympathetic activation was associated with a decrease in cardiorespiratory interactions and the RSA-loop during the head-up tilt test [100], confirming the assumption that SZO were associated with higher sympathetic activation. The nonlinear cardiorespiratory information transfer was significantly reduced in both directions (BBI→RESP, RESP→BBI) indicating that, in general, nonlinear regulatory processes are inhibited. In the study of Peupelmann et al. [16], they could show that the severity of schizophrenia is associated with breathing patterns assuming that an inhibition of vagal control centers at the brainstem are responsible for their findings. The causal information flow RESP→BBI independent of arterial pressure changes characterizes central respiratory driving mechanisms related to alterations in heart rate on the cardiac vagal motor neurons [101]. Central respiratory driving mechanisms seem to be inhibited and directly connected to alterations in the cardiac regulatory system in SZO [19]. It was demonstrated that the frontal, central (investigated in this study) and occipital brain regions are involved in central-cardiorespiratory information transfers which are altered to physiological conditions such as wake and sleep [1].

Linear and nonlinear brain-heart information flows (P_EEG_→BBI) were characterized as top-down from the CNS (brain) towards ANS (heart) in both groups. It leads to the assumption that an impaired brain-heart axis is present in schizophrenia, and more expressed in the linear domain and here more in the direction autonomic to central regulation. A functional disconnection between the CNS, impaired interactions of fronto-cingulate and subcortical brain regions, and ANS was suggested for SZO, when these patients process threat-related signals [102]. Impairments in frontal-subcortical processes are associated with psychopathologies such as schizophrenia [103]. Internally generated processes of misjudgments of threat-related signals like anxiety seem to be associated with paranoid cognition due to a breakdown of these processes. This leads to an inhibited central-cardiac coupling influenced by a lack of cortical inhibitory control over sympathetic-excitatory subcortical regions [102]. Moreover, it was hypothesized that the inhibitory deficit was reflected in impaired cognitive and behavioral inhibition connected with an impaired HRV [104,105]. 

The linear bidirectionally directed central-respiratory information transfer (P_EEG_↔RESP(BBI)) was dominated towards CNS from respiration. Due to, the respiratory pathway being more pronounced instead of the cardiac one. This leads to the assumption that the closed-loop of central-cardiorespiratory information transfer is less pronounced by central driving on the respiratory system to adapt heart rate but stronger by the ANS. However, it seems that central-cardiorespiratory feedback-loop in the direction from ANS towards CNS is strongly dominated by respiration that functions as a feedback trigger to central regulatory processes for more information transfer towards the ANS for adaptation oxygenation. The brainstem and higher brain centers (limbic system, cortical structures) interacting to maintain the final respiratory output, mainly regulated for metabolic and homeostatic purposes and altered in reaction to emotions [85] such as fear and anxiety present in SZO. 

It was stated by Williams et al. [102] that a disassociation of amygdala-prefrontal process circuits and arousal leads to an inhibition of the signal processing of threat-related signals in SZO. Particularly, the dysregulation in the normal cycle of mutual feedback between the amygdala and autonomic regulatory activity is characterized by reduced amygdala activity and excessive arousal in these patients. The medial prefrontal area responsible for maintaining amygdala-autonomic working processes seem to be not able to perform its function resulting in a perseveration and exacerbation of arousal responses (threat-related signals (skin conductive response)). 

For schizophrenia there is a continuing debate what the defined reasons for the dysregulation of the ANS, and thereby, the impaired brain-heart coupling. It is suggested that antipsychotic medications which suppress dopamine activity in the mesolimbic pathway of the brain, genetics and neurobiological processes are important contributory factors also because different brain areas (cortical, subcortical, brainstem) are involved in autonomic regulation. The dynamically interacting network of physiological systems and subsystems within the human body are connected in a close way, that a failure of one system or subsystem can lead to a chain of faults and thus impairing the dynamical interplay within the whole network [106]. For SZO it has been shown that autonomic dysfunction is closely associated with deficits of prefrontal cortex activity in executive function and inhibition [104,105]. It has been suggested [104] that the lack of inhibition of amygdala mediating cardiovascular and autonomic responses to stress by the prefrontal cortex is one reason for ANS dysregulation. The meta-analysis by Thayer et al. [107] showed that HRV can indicate the degree to which a medial prefrontal cortex guided “core integration” directly regulates the heart. The medial prefrontal cortex is involved in the regulation of both behavioral and physiological responses, including the regulation of anxiety, heart rate changes associated with social threat, and a variety of other peripheral responses to stressors associated with the brain stem regulatory function. It has been shown that the left and right hemispheres are responsible for parasympathetic and sympathetic cardiovascular regulation processes and that change in functional activation of the cerebral system and changes in heart rate and blood pressure are related [96].

Limitations that have to be addressed are: (1) the treatment with antipsychotic drugs as a standard therapeutic measure, and (2) no comparative fMRI analyses were performed to prove which parts of the frontal cortex are involved in cerebral activation of the cortical as well as subcortical centers. As an outlook it seem to be very promising to combine fMRI and EEG analysis to get new perspectives in cognitive functions in respect to the central-autonomic-network in SZO [108]. The objective of this manuscript was to evaluate the general behavior of the underlying short-term couplings and an average of features (windows) over time was performed (no real-time analyses). From the methodical aspect, it would be interesting in an ongoing study if other coupling approaches (e.g., time-invariant approach based on Granger causality, recurrence quantification analysis, functional connectivity analysis approaches) would reveal new insight in central-autonomic-network pathways and possibly would have a greater discriminative effect [44,45,109]. 

## 5. Conclusions

This study provides new insights within central-autonomic network pathways in respect to central and cardiovascular-cardiorespiratory regulation processes in schizophrenia. In sum, we could demonstrate significantly weaker nonlinear central-cardiovascular and central-cardiorespiratory coupling pathways, and significantly stronger linear central information flow in the direction of the cardiac- and vascular system, and a significantly stronger linear respiratory information transfer towards the central nervous system in schizophrenia. The detailed findings how the different pronounced central-autonomic network pathways are associated with paranoid schizophrenia might allow a better understanding how cerebral activation and autonomic responses and/or activation are connected in physiology networks under pathophysiological conditions. Further studies are of course required.

## Figures and Tables

**Table 1:** Indices from heart rate variability (HRV), blood pressure variability (BPV), respiratory variability (RESPV), and electroencephalogram (EEG) which discriminates between paranoid schizophrenia patients (SZO) and healthy subjects (CON).

	Index	CON			SZO		
		mean	±	std	mean	±	std
**HRV**	meanNN_BBI_	904.2	±	153.0	709.4	±	104.7 ***
	sdNN_BBI_	52.0	±	23.0	32.3	±	23.4 *
**BPV**	meanNN_SYS_	134.9	±	19.8	121.4	±	15.4 *
	sdNN_SYS_	9.2	±	3.0	10.0	±	6.8
	meanNN_DIA_	69.8	±	12.8	66.7	±	12.2
	sdNN_DIA_	0.8	±	0.9	2.5	±	3.6
**RESPV**	meanNN_RESP_	4.0	±	1.1	3.7	±	0.8
	sdNN_RESP_	0.7	±	0.6	0.7	±	0.4
	BF	16.2	±	3.0	17.7	±	3.5
**EEG**	P	820.6	±	860.8	357.8	±	732.8 ***

(* *p* < 0.05; *** *p* < 0.0022; n.s. = not significant).

**Table 2:** Linear (NSTPDC) central-cardiovascular (BBI, SYS, and P_EEG_) coupling analyses results to discriminate between patients suffering from paranoid schizophrenia (SZO) and healthy subjects (CON).

	Index	CON			SZO		
		mean	±	std	mean	±	std
**BBI** ↔ **SYS**	NF	−0.66	±	0.52	−0.48	±	0.81 ***
	A_BBI→SYS(PEEG)_	0.25	±	0.06	0.27	±	0.14
	A_SYS→BBI(PEEG)_	0.43	±	0.14	0.39	±	0.16 ***
**BBI** ↔ **P_EEG_**	NF	−0.64	±	0.86	−0.81	±	1.03 **
	A_BBI→PEEG(SYS)_	0.10	±	0.05	0.09	±	0.06 *
	A_PEEG→BBI(SYS)_	0.23	±	0.16	0.26	±	0.17 *
**SYS** ↔ **P_EEG_**	NF	0.00	±	1.07	−0.70	±	0.94 ***
	A_SYS→PEEG(BBI)_	0.13	±	0.07	0.10	±	0.06 ***
	A_PEEG→SYS(BBI)_	0.14	±	0.10	0.20	±	0.13 ***

(BBI: beat-to-beat intervals; SYS: end-systolic blood pressure amplitude values over time; P_EEG_: the mean power in the BBI-related EEG intervals; * *p* < 0.05; ** *p* < 0.01; *** *p* < 0.0022; n.s. = not significant; # = not confirmed by surrogate analysis).

**Table 3:** Nonlinear (MuTE) central-cardiovascular (BBI, SYS, and P_EEG_) coupling analyses results to discriminate between patients suffering from paranoid schizophrenia (SZO) and healthy subjects (CON).

	Index	CON			SZO		
		mean	±	std	mean	±	std
**BBI** ↔ **SYS**	BBI→SYS(P_EEG_)	0.098	±	0.035	0.064	±	0.042 ***
	SYS→BBI(P_EEG_)	0.053	±	0.034	0.093	±	0.037 ***
**BBI** ↔ **P_EEG_**	BBI→P_EEG_(SYS)	0.012	±	0.011	0.007	±	0.009 ***
	P_EEG_→BBI(SYS)	0.012	±	0.009	0.007	±	0.008 ***
**SYS** ↔ **P_EEG_**	SYS→P_EEG_(BBI)	0.012	±	0.011	0.006	±	0.008 ***
	P_EEG_→SYS(BBI)	0.008	±	0.008	0.006	±	0.008 ***

(BBI: beat-to-beat intervals; SYS: end-systolic blood pressure amplitude values over time; P_EEG_: the mean power in the BBI-related EEG intervals; *** *p* < 0.0022; n.s. = not significant; # = not confirmed by surrogate analysis).

**Table 4:** Linear (NSTPDC) central-cardiovascular (BBI, RESP, and P_EEG_) coupling analyses results to discriminate between patients suffering from paranoid schizophrenia (SZO) and healthy subjects (CON).

	Index	CON			SZO		
		mean	±	std	mean	±	std
**BBI** ↔ **RESP**	NF	−1.56	±	0.34	−1.48	±	0.69
	A_BBI→RESP(PEEG)_	0.05	±	0.02	0.04	±	0.03 ***
	A_RESP→BBI(PEEG)_	0.25	±	0.08	0.27	±	0.17
**BBI** ↔ **P_EEG_**	NF	−0.48	±	0.76	−0.13	±	0.91 ***
	A_BBI→PEEG(RESP)_	0.10	±	0.05	0.12	±	0.06 ***
	A_PEEG→BBI(RESP)_	0.16	±	0.08	0.15	±	0.07 *
**RESP** ↔ **P_EEG_**	NF	0.99	±	0.66	1.26	±	0.62 ***
	A_RESP→PEEG(BBI)_	0.19	±	0.07	0.24	±	0.11 ***
	A_PEEG→RESP(BBI)_	0.06	±	0.03	0.05	±	0.03 ***,#

(BBI: beat-to-beat intervals; RESP: respiratory frequency; P_EEG_: the mean power in the BBI-related EEG intervals; * *p* < 0.05; *** *p* < 0.0022; n.s. = not significant; # = not confirmed by surrogate analysis).

**Table 5:** Nonlinear (MuTE) central-cardiovascular (BBI, RESP, and P_EEG_) coupling analyses results to discriminate between patients suffering from paranoid schizophrenia (SZO) and healthy subjects (CON).

	Index	CON			SZO		
		mean	±	std	mean	±	std
**BBI** ↔ **RESP**	BBI→RESP(P_EEG_)	0.020	±	0.013	0.015	±	0.012 ***
	RESP→BBI(P_EEG_)	0.033	±	0.009	0.026	±	0.012 ***
**BBI** ↔ **P_EEG_**	BBI→P_EEG_(RESP)	0.014	±	0.011	0.012	±	0.011 *
	P_EEG_→BBI(RESP)	0.016	±	0.010	0.014	±	0.010
**RESP** ↔ **P_EEG_**	RESP→P_EEG_(BBI)	0.017	±	0.010	0.014	±	0.009 ***
	P_EEG_→RESP(BBI)	0.015	±	0.008	0.012	±	0.009 ***

(BBI: beat-to-beat intervals; RESP: respiratory frequency; P_EEG_: the mean power in the BBI-related EEG intervals; * *p* < 0.05; *** *p* < 0.0022; n.s. = not significant; # = not confirmed by surrogate analysis).
